# Alzheimer’s disease: insights from a network medicine perspective

**DOI:** 10.1038/s41598-022-20404-3

**Published:** 2022-10-07

**Authors:** Federica Conte, Paola Paci

**Affiliations:** 1 Institute for Systems Analysis and Computer Science “Antonio Ruberti”, National Research Council, Rome, Italy; 2 Present Address: Department of Computer, Control and Management Engineering, Sapienza University of Rome, Rome, Italy

**Keywords:** Computational biology and bioinformatics, Biomarkers, Alzheimer's disease

## Abstract

Alzheimer’s disease (AD) is the most common neurodegenerative disease that currently lacks available effective therapy. Thus, identifying novel molecular biomarkers for diagnosis and treatment of AD is urgently demanded. In this study, we exploited tools and concepts of the emerging research area of Network Medicine to unveil a novel putative disease gene signature associated with AD. We proposed a new pipeline, which combines the strengths of two consolidated algorithms of the Network Medicine: DIseAse MOdule Detection (DIAMOnD), designed to predict new disease-associated genes within the human interactome network; and SWItch Miner (SWIM), designed to predict important (switch) genes within the co-expression network. Our integrated computational analysis allowed us to enlarge the set of the known disease genes associated to AD with additional 14 genes that may be proposed as new potential diagnostic biomarkers and therapeutic targets for AD phenotype.

## Introduction

Alzheimer’s disease (AD) is a progressive neurodegenerative disorder estimated to affect nearly 30 million people worldwide^1^. AD is the most common cause of dementia in mid- and late-life, but its clinical impact is modified by other neurodegenerative and cerebrovascular conditions (e.g., impairment in memory, judgment, decision making, distorted language, and physical orientation)^2,3^. Over the last years, significant advances were made in the understanding of AD by investigating the molecular mechanisms underlying amyloid-β peptides and protein tau, both hallmarks of this disease. Despite this progress, no disease-modifying treatments currently exist in AD. Indeed, the amyloid-β and tau biomarkers are very useful in tracking AD progression but, unfortunately, they have not yet translated into treatments for AD. Thus, the identification of novel and effective molecular biomarkers for the early diagnosis and treatment of AD is urgently demanded.

In this perspective, fundamental insights may come from the new emerging research area of Network Medicine, which exploits concepts garnered from network theory to elucidate the relation between perturbations in the human interactome (i.e., the network of all physical interactions within a cell, from protein–protein to regulatory protein–DNA and metabolic interactions) and phenotypic disease manifestations^4^. In particular, an extensive literature on this topic demonstrated that the molecular determinants associated with a given disease (named disease genes) are not randomly scattered, but form locally dense and topologically/functionally well-defined regions in the human interactome (named disease modules)^5–10^. More precisely, the fundamental hypotheses of Network Medicine state that the molecular determinants associated with a specific disease: (i) have an increased tendency to interact with each other (local hypothesis); (ii) show a tendency to cluster in the same network neighbourhood (disease module hypothesis); (iii) show a tendency to be involved in closely disease-related cellular functions or causal molecular pathways (functional coherence hypothesis)^4,7^. These observations have fueled the development of numerous algorithms to interrogate disease etiology, model molecular and genetic interactions, identify potential biomarkers, and design therapeutic interventions^9^. Such algorithms were highly customized according to the study design, the phenotype under investigation, the biological question of interest, the molecular entities measured, and the type and size of the available datasets. Several of these algorithms^10–12^ make use of the human interactome, also denoted as protein–protein interaction (PPI) network, and of the a priori knowledge of disease genes defined as genes with mutations known to have a phenotypic impact^13,14^. Among them, one of the most well-established algorithm is DIAMOnD (DIseAse MOdule Detection)^12^, which was designed to predict the full disease module around a set of known disease associated proteins (called seed proteins) in the human interactome by performing a systematic analysis which prioritizes the proteins having a significant fraction of their interactions with the seed proteins. Other algorithms make use of gene expression networks (GENs) that are built by calculating correlations between the expression profiles of each gene pair. Among them, one of the most promising algorithm is SWIM (SWItch Miner)^15^, which was designed to predict important (switch) genes within GENs that are associated with intriguing patterns of molecular co-abundance and may play a key role in phenotypic transitions in various biological settings^2,15–18^. In particular, switch genes are defined as a special set of genes with peculiar topological features within GENs, i.e.: (i) they show coherent patterns of correlation, suggesting they may be co-regulated or functionally related; (ii) they form localized connected subnetworks/modules; (iii) they are not local hub within their module, but they act as connectors able to convey information among modules of the correlation network.

Summarizing, while PPI networks leverage experimentally confirmed interactomics data pointing out physical and functional interactions occurring among proteins, GENs leverage phenotype-specific gene expression data resulting in a context-specific gene interaction network.

Here, to combine the strengths of both methodologies, we integrated the DIAMOnD and SWIM results by mapping the list of the AD-specific switch genes in the subnetwork of the human interactome constituted by the nearest neighbours of the AD-associated seed proteins.

Regarding AD gene expression data, there exist several publically available datasets. For example, Liang et al. collected transcriptomic data coming from post-mortem brain tissue and identified region-specific gene expression alterations in AD brains^19^. In this study, we decided to pursue a blood-based gene expression analysis since the identification of a blood-based diagnostic signature could be extremely useful for pre-screening ahead of invasive and costly follow-up analysis^20–22^. Indeed, blood-based biomarkers represent an ideal option as the first-step of the diagnostic process beginning in primary care settings, and may help to determine which individuals should receive a referral to assessment by specialists, including CSF analysis, MRI or amyloid PET diagnostics. Another advantage is that blood testing is already a well-established part of clinical routines, requiring no further training for health care professionals. In addition, the use of blood-based biomarkers may offer the possibility to test a wide range of other candidate pathophysiological biomarkers, reflecting the full spectrum of disease and driving molecular mechanisms underlying AD, beyond the standard amyloid- and tau-based tests^21^.

Our network medicine approach led to an in silico recognition of a novel disease gene signature for AD composed of proteins (14 switch genes plus the already known 99 AD-associated seed proteins) having unique, quantifiable characteristics that distinguish them from the other nodes in the human interactome: they are physically and functionally related and AD phenotype-specific in the human interactome.

We believe that our findings could provide advancements in the ongoing effort to identify effective diagnostic biomarkers and therapeutic targets for AD so to improve the clinical management of this dreadful disease.

## Materials and methods

### Alzheimer’s disease gene expression data

The AD dataset is available through the GEO public repository at accession numbers GSE63060 (batch 1) and GSE63061 (batch 2) published on August 05, 2015 and updated on May 03, 2019^20^. As already described in^7^, data include expression profiling by array related to Alzheimer’s Disease (AD) and control samples (CTL) originating from the EU funded AddNeuroMed Cohort^23^, which is a large cross-European AD biomarker study relying on human blood as the source of RNA. In particular, GSE63060 comes from array A-MEXP-1171—Illumina HumanHT-12 v3.0 Expression BeadChipm and includes a total of 249 samples (145 AD, 104 CTL); whereas GSE63061 comes from array A-GEOD-10558—Illumina HumanHT-12 V4.0 expression beadchip and includes a total of 273 samples (139 AD and 134 CTL). The probe-sets were mapped to official gene symbols using the relative platform (GPL6947-13512 for GSE63060 and GPL10558-50081 for GSE63061) available from the GEO repository. Multiple probe measurements of a given gene were collapsed into a single gene measurement by considering the mean. By matching genes based on gene symbols, we created a single merged dataset with both batches and we used Combat function from R/Bioconductor package SVA to correct for batch-specific effects. Finally, we obtained a data matrix of 19,460 gene symbols (rows) and 522 samples (columns) including 284 AD and 238 CTL. Table 1 reports all available information for AD case–control samples of the gene expression dataset under study.

**Table 1:** AddNeuroMed dataset.

	Total samples	Gender	Age (mean ± SD)
control	238	143 F (60%); 95 M (40%)	72 ± 19.8 yr
AD case	284	184 F (65%); 100 M (35%)	70 ± 17 yr

AD, Alzheimer’s disease; F, Female; M, Male; sd, standard deviation; yr, years.

### Human interactome and Alzheimer’s disease associated genes

The human interactome was downloaded from Ghiassian and coauthors^12^ that assembled direct physical protein interactions with reported experimental evidence (e.g., binary interactions, literature curated interactions, metabolic enzyme-coupled interactions) from several consolidated data sources such as IntAct^24^ MINT^25^, and BioGRID^26^. The union of all these interactions yields a network of 13,460 proteins that are interconnected by 141,296 physical interactions.

For the disease-gene associations, we considered the 29 Alzheimer’s disease associated genes collected by Ghiassian and coauthors^12^ and derived from OMIM (Online Mendelian Inheritance in Man) database^27^ and GWAS (Genome-Wide Association Studies) database^13^. In addition, we also included the findings of the very recent and fundamental study by Bellenguez and colleages^28^, where the authors performed a genome-wide association study totaling 111,326 clinically diagnosed/‘proxy’ AD cases and 677,663 controls. This study led to the identification of 75 risk loci corresponding to a list of 77 GWAS genes , which compasses 7 GWAS genes (i.e., CR1, BIN1, TREM2, CD2AP, PHA1 CLU, APP) collected by Ghiassian et al.^12^. The union of the two lists collected by Ghiassian et al.^12^ and identified by Bellenguez et al.^28^ ended up with a unique pool of 99 AD-associated GWAS genes (Supplementary Table 1).

### DIAMOnD tool

**Figure 1:**
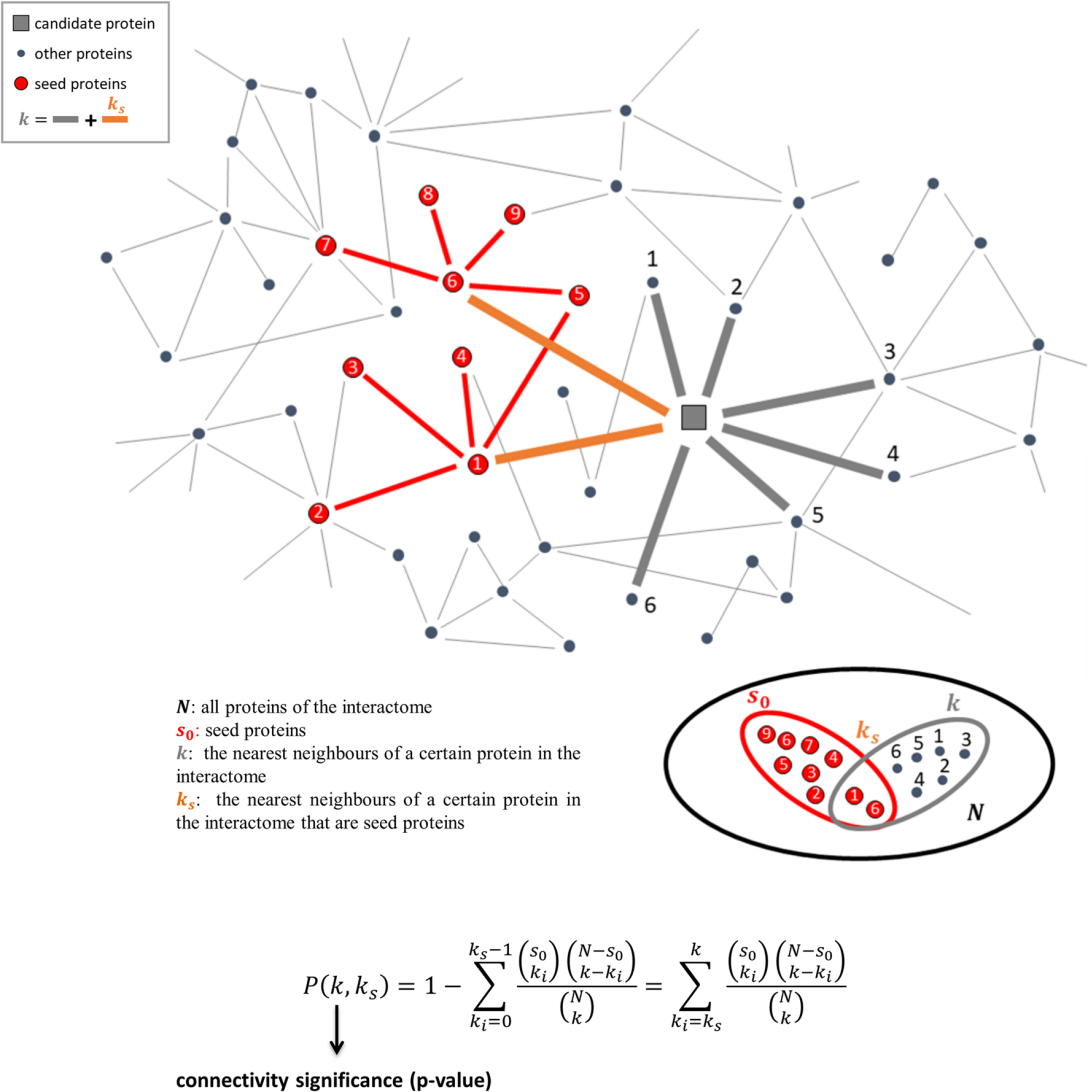
DIAMOnD methodology. The network corresponds to the human interactome where the orange circular nodes are the seed proteins, the green square node is the protein to test with k connections (red and grey thick links) including k_s_ links to seed proteins (red thick links), the grey circular nodes refer to other proteins in the interactome. The ensembles considered in the hypergeometric test for the computation of connectivity significance are reported.

In order to predict novel disease genes for AD, we exploited the network-based DIAMOnD (Disease Module Detection) tool^12^. DIAMOnD implements a series of well-defined steps described in details in^12^. Specially, DIAMOnD starts from the seed ${\mathrm{s}}_{0}$ and uses the hypergeometric distribution to calculate the probability of having drawn ${\mathrm{k}}_{\mathrm{s}}$ seed proteins (out of $\mathrm{k}$ total draws) from a population of $\mathrm{N}$ proteins (Fig. 1). In the network perspective, the $\mathrm{N}$ proteins are the nodes of the human interactome and $\mathrm{k}$ are the nearest neighbours of a certain protein in the network (Fig. 1). Through an iterative procedure, DIAMOnD computes, for all proteins of the interactome with at least one link with the seed, the so-called connectivity significance (CS). The CS represents the probability (*p* value) that a protein with a total of $\mathrm{k}$ links has ${\mathrm{k}}_{\mathrm{s}}$ or more connections to seed proteins than expected (Fig. 1) and it was used to prioritize the proteins. In particular, the protein with the highest rank (i.e. lowest *p* value or CS) becomes a “candidate protein” and it is added to the set of seed nodes, increasing their number from ${\mathrm{s}}_{0}$ to ${\mathrm{s}}_{1}= {\mathrm{s}}_{0}+1$. This procedure is iterated for a fixed number of iterations (i.e., 500) by expanding the disease module by one node at each iteration step. Thus, at the end of the 500 fixed iterations, the algorithm provides a ranked list of proteins (as many as the iterations performed), each with its own associated connectivity significance (*p* value). The order in which the DIAMOnD predictions are pulled into the disease module reflects their topological relevance to the disease.

### Biological evaluation of DIAMOnD predictions

In order to select the most promising disease protein candidates predicted by DIAMOnD, the so-called biological criterion was exploited^12^. This criterion is based on the hypothesis that proteins with biological annotations similar to the ones of the seed proteins of a given disease are more likely to be associated to that disease. The biological evaluation of DIAMOnD predictions exploits a sliding window approach. In particular, a sliding window, whose size is equal to the number of seed proteins, is defined and a functional enrichment analysis is performed to identify the biological annotations (e.g. GO terms or KEGG pathways) enriched in the list of the initial seed proteins. Candidate proteins within the sliding window that are annotated with any of the enriched biological annotations are considered true positives. The sliding window span across the list of DIAMOnD predicted proteins and at each iteration step, a hypergeometric test is performed to evaluate if the number of true positives is greater than what expected by chance. Thus, the application of this hypergeometric test provides a *p* value (called enrichment *p* value) associated with each iteration of the DIAMOnD algorithm. The maximal number of DIAMOnD predicted proteins that should be considered corresponds to the number of iterations such that the number of DIAMOnD proteins with direct statistically significant biological evidence reaches a plateau (enrichment *p* value ≤ 0.01, where 0.01 is the threshold of the enrichment *p* value chosen in the present study). The functional enrichment analysis of the initial seed proteins was conducted by considering the KEGG pathways annotations provided by Enrichr web tool and updated to 2021^29^. KEGG pathways were considered statistically significant with an adjusted *p* value ≤ 0.05.

### SWIMmeR tool

In order to identify AD-specific switch genes, we exploited the network-based SWIMmeR tool^30^, an R open-source version of its predecessor SWIM (SWitchMiner) written in MATLAB®^15^. SWIMmeR (and SWIM) implements a series of well-defined steps described in details in^15,30^. Specially, SWIMmeR starts from the computation of differentially expressed genes (DEGs) between two condition of interest (e.g. physiological *versus* pathological condition) and uses DEGs to build a correlation network where two nodes are connected if the absolute value of the Pearson correlation coefficient of their expression profiles is greater than a given threshold. This threshold should be selected in order to have a manageable network as well preserve the integrity of the network. Next, SWIMmeR identifies communities in the correlation network by means of the k-means clustering algorithm. Then, it searches for specific topological properties of the correlation network using the date/party/fight-club hub classification^15^, based on the Average Pearson Correlation Coefficients (APCCs) between the expression profiles of each hub (i.e., node with degree greater than 5) and its nearest neighbors. Finally, SWIMmeR assigns a role to each node based on its ability to convey information within and between clusters in the network through the computation of two parameters: the within-module degree, which quantifies how much a node is a hub in its own cluster, and thus, it is a measure of local connectivity; and the clusterphobic coefficient, which quantifies the ratio of internal to external connections of a node, and thus, it represents a measure of global connectivity. Switch genes are identified as a special subclass of fight-club hubs (i.e., hubs with negative APCC values) interacting mainly outside their own cluster. All the parameters used in this study for SWIMmeR run are reported in Table 2.

**Table 2:** Summary of SWIMmeR parameters.

SWIMmeR run for AD dataset	
Number of control samples	238
Number of AD case samples	284
FC threshold	1
FDR threshold	0.001
Number of DEGs	2327
PC threshold	0.46 (83th prc)
Numeber of network nodes	2249
Number of clusters	3
Number of switch genes	598

AD, Alzheimer’s disease; FC, fold-change; FDR, False Discovery Rate; DEGs, Differentially Expressed Genes; PC, Pearson Correlation.

### Identification of disease modules

In order to test whether a given list of genes forms a statistically significant disease module, we exploited the procedure firstly proposed in^7^. Specifically, the genes were mapped onto the human interactome, the corresponding subnetwork was extracted, and the following three metrics were computed: (1) the size of the largest connected component (LCC); (2) the number of interactions in the LCC; and (3) the total number of interactions (edges). In order to complement these metrics with a measure of statistical significance, we evaluated the probability that the given list of genes was localized within a certain network neighborhood greater than expected by chance^7^. To do so, we randomly selected groups of proteins in the human interactome with the same size and degree distribution as the original list of genes and we computed the three above-mentioned metrics. This procedure was repeated 1,000 times, and we derived three distributions for all three metrics corresponding to the subgraph induced by the random gene set. The three metrics calculated for the original list of genes were z-score-normalized with respect to the corresponding reference random distribution. Subsequently, the *p* value for the given z statistic was calculated, expecting a *p* value ≤ 0.05 for genes forming a statistically significant disease module^7^.

### Functional enrichment analysis

The functional enrichment analysis was performed by querying KEGG^31^, GO Gene Ontology^32^ and DisGeNET^2^ though Enrichr web tool^29^. *p* values were adjusted with the Benjamini–Hochberg method and a threshold equal to 0.05 was set to identify functional annotations and disease-gene associations significantly enriched amongst the selected genes.

## Results

### Study design

**Figure 2:**
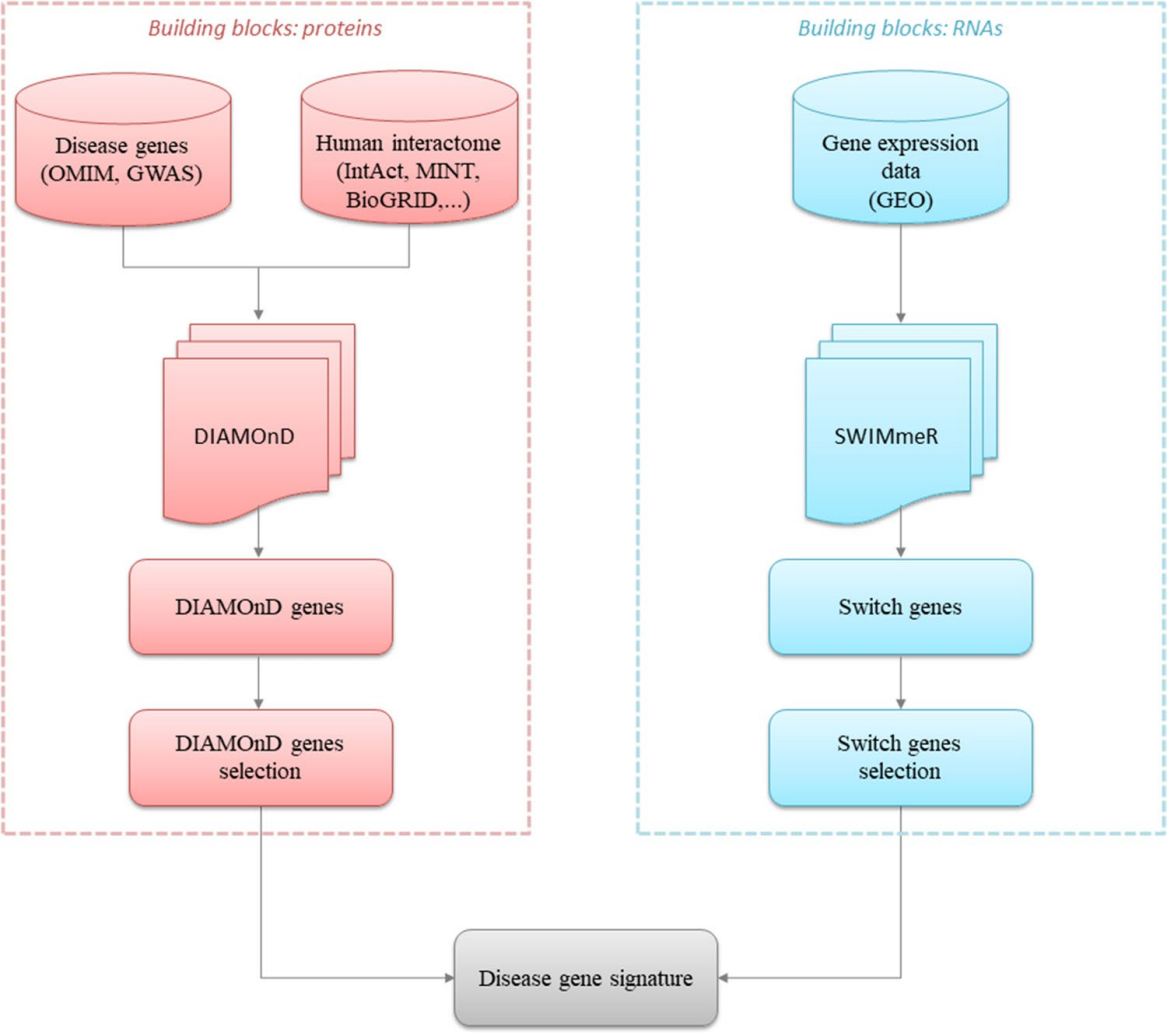
Study design. The figure depicts the schematic of the analysis applied in this study.

In the present study, we aim to propose a novel disease gene signature associated with Alzheimer’s disease by integrating two well-established Network Medicine methodologies, i.e. DIAMOnD and SWIM (in particular its R implementation called SWIMmeR). A schematic of our study design is depicted in Fig. 2.

### Identification of DIAMOnD genes

The DIAMOnD methodology was applied to enlarge the set of 99 known AD-associated genes (referred as seed) in the human interactome, offering a ranked list of 500 new candidate disease genes (refereed as DIAMOnD genes) having a significant fraction (connectivity significance ranging from 9.62E-22 to 3.52E-05) of their interactions with the seed (Supplementary Table 1).

**Figure 3:**
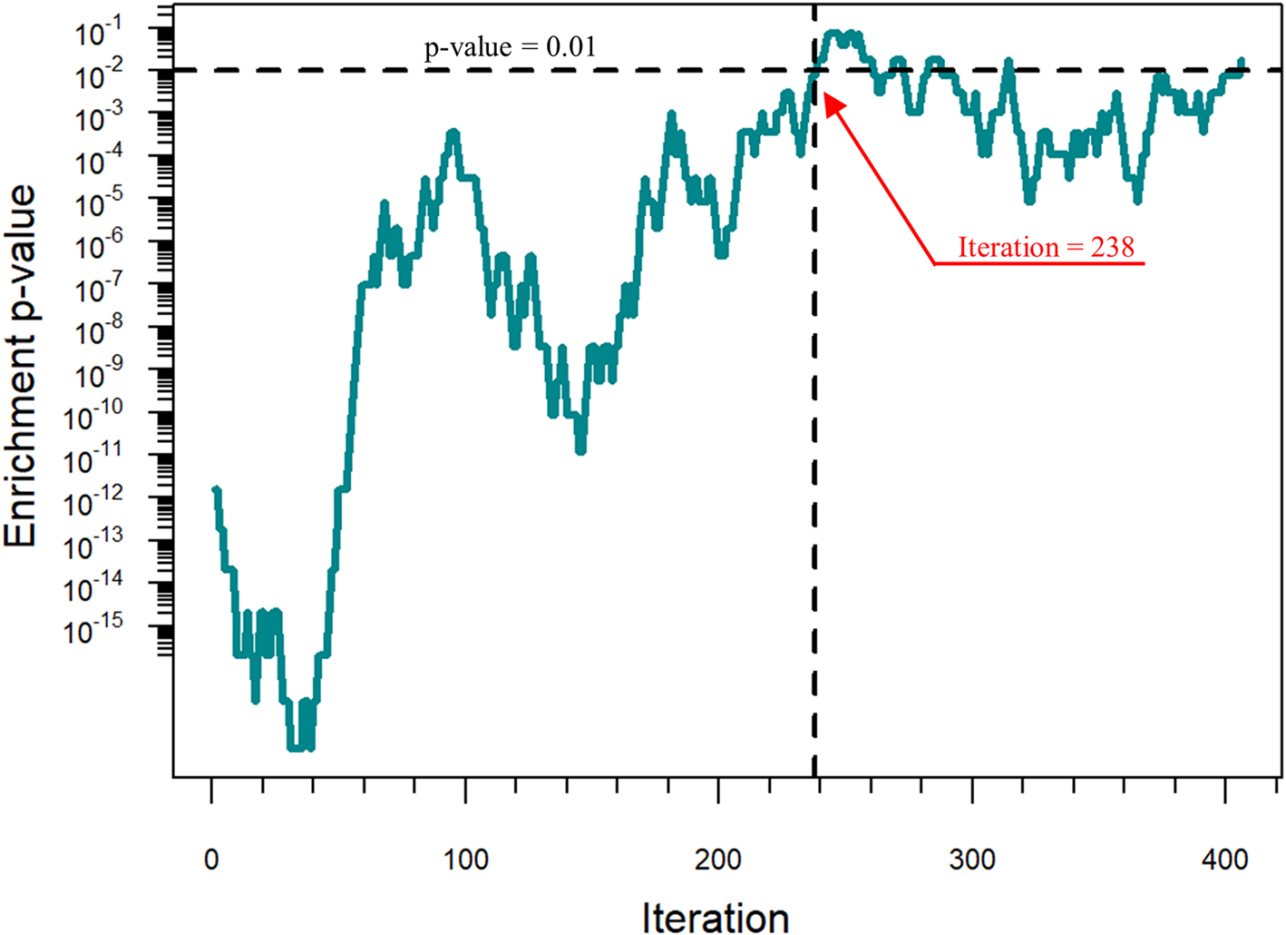
Biological evaluation of DIAMOnD predictions. The significance (enrichment *p* value ≤ 0.01) of the similarity between DIAMOnD predicted disease genes and seed genes suggests a cutoff corresponding to the iteration 238.

This approach, however, requires an additional criterion to define the boundary of the AD disease module, i.e. a threshold for the total number of novel candidates to be considered. The connectivity significance (*p* value) cannot be used directly to define this threshold since the disease module grows at each iteration step and then the number of seed proteins on which the *p* value is based increases (see Materials and Methods). Since larger sets can produce smaller p- values, the absolute significance values obtained at different iteration steps cannot be compared to each other. Thus, in order to select the most promising DIAMOnD predictions, we exploited the so-called biological criterion proposed by^12^ and based on the assumption that candidates with biological characteristics similar to the ones of the initial seed are more likely to be disease associated as well (see Materials and Methods). We found that the DIAMOnD genes with direct biological evidence (enrichment *p* value ≤ 0.01) are the first 238, suggesting this as the maximal number to be considered (Fig. 3). This criterion allowed to identify 238 DIAMOnD predicted genes for AD (Supplementary Table 1).

### Identification of switch genes

The SWIM methodology was applied on the blood-based gene expression profiles of 284 AD samples and 238 control samples (Table 2) freely available from GEO database (see Materials and Methods) to extract the AD-specific switch genes.

**Figure 4:**
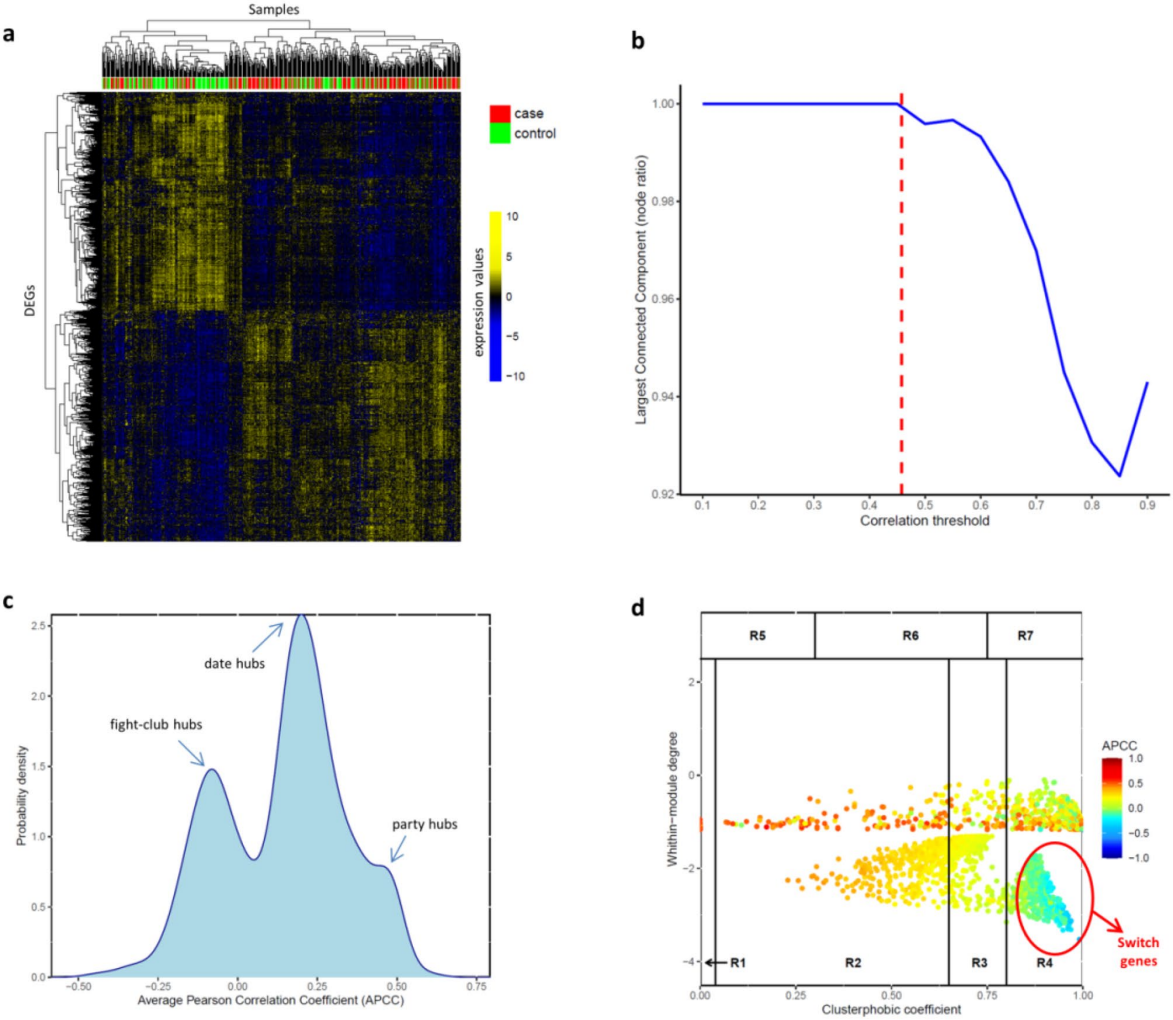
SWIMmeR results on AD gene expression data. (**a**) Differentially expressed genes (DEGs). Differentially expressed profiles are clustered according to genes (rows) and samples (columns) by using Pearson correlation distance as metrics. Heat map colours represent different expression levels increasing from blue to yellow. AD samples (case) are indicated in red, whereas control samples are indicated in green. (**b**) Connectivity of correlation network. The x-axis represents the Pearson correlation threshold varying in the chosen range, while the y-axis represents the fraction of nodes populating the largest component. The dashed red line corresponds to the selected threshold (i.e., 0.46 or 83th percentile). Note that y = 1 means that all nodes fall in the largest component and thus the network is fully connected; otherwise more components exist. (**c**) APCC distribution. Probability distribution of APCC for hubs (i.e., node with degree greater than 5) identified in the AD correlation network. Note the presence of the three peaks associated to the date/party/fight-club hub classification. (**d**) Heat cartography map. Dots correspond to nodes in the AD correlation network. Each node is coloured according to the value of the APCC between its expression profile and that of its nearest neighbours in the network.

Firstly, SWIMmeR identified 2327 genes showing a statistically significant differential expression (FDR < 0.01) between the AD and control condition (Fig. 4a and Supplementary Table 2). The differentially expressed genes (DEGs) were then used to build the AD correlation network. In this study, we selected a correlation threshold corresponding to the 83th percentile (Table 2) of the entire correlations’ (Fig. 4b). Next, SWIMmeR identified three clusters in the AD correlation network (Supplementary Figure 1a) and computed APCC distribution, revealing the three peaks associated with the date/party/fight-club hub classification^15^ (Fig. 4c). Finally, SWIMmeR assigned a role to each network node based on the values of the within-module degree and the clusterphobic coefficient in order to unveil the special set of switch genes (Fig. 4d).

The SWIM-based analysis of the AD gene expression dataset led to the identification of 598 switch genes, including 584 (98%) up-regulated and 14 (2%) down-regulated genes (Supplementary Table 2). Among them, we focused on those switch genes showing a coherent gene expression pattern and falling in the same network cluster, i.e. the 448 switch genes up-regulated in the AD condition and included in cluster 1 of the correlation network built by SWIMmeR (Supplementary Figure 1b and Supplementary Table 2). Notably, five of these switch genes (i.e. GBA, BCKDK, GRN, SORL1, RBCK1) are known disease genes of AD. Moreover, by performing a functional enrichment analysis, we found that these switch genes are significantly involved in several inflammatory and innate immune mechanisms (Supplementary Table 3) able to promote Alzheimer's disease according with a large body of evidence^33–36^.

### Recognition of AD gene signature

**Figure 5:**
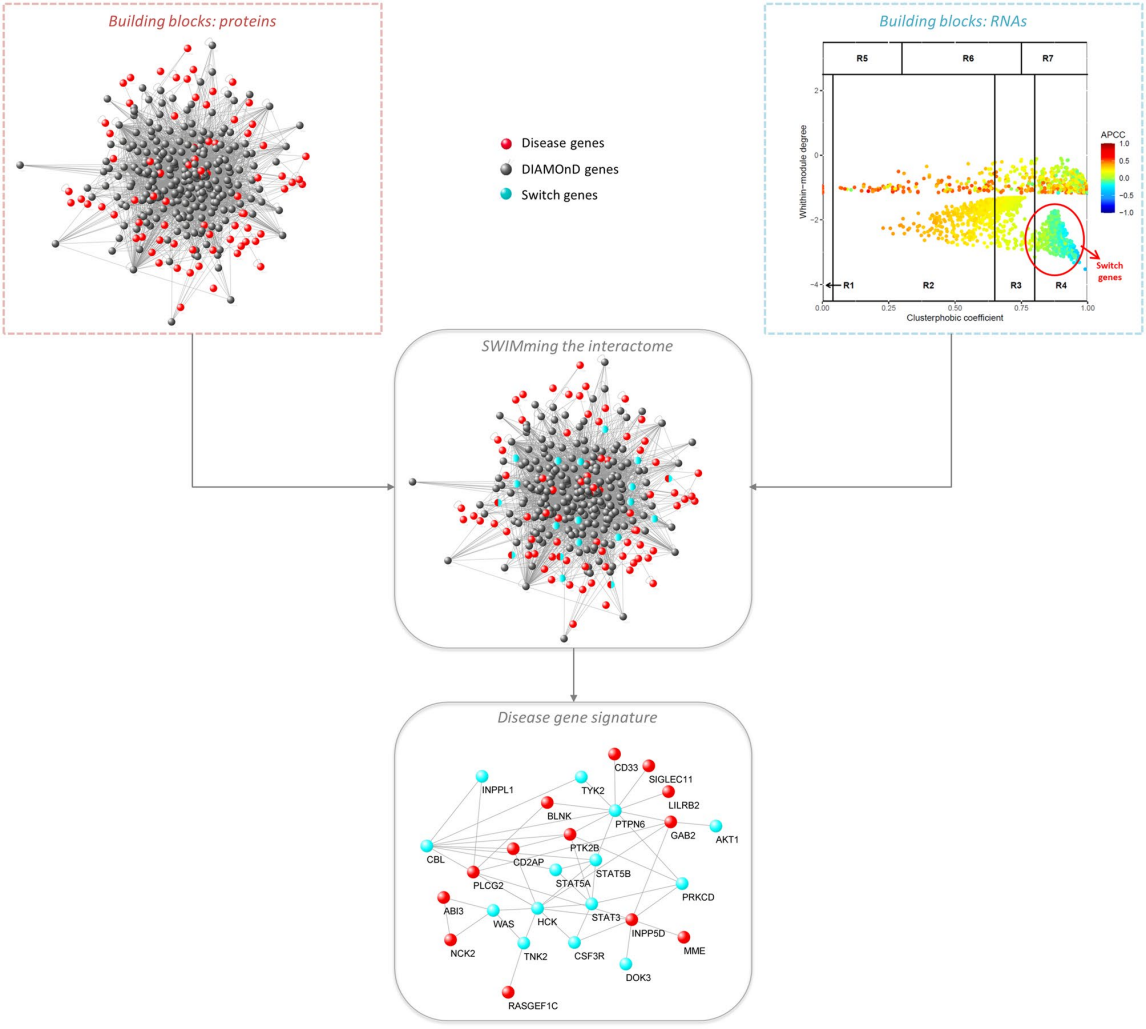
AD gene signature. By integrating the DIAMOnD and SWIMmeR results, the set of the known disease genes associated to AD (seed) was enlarged with additional 14 genes that are switch genes and that physically interact with each other and with the seed.

Starting from the known AD-associated genes (seed proteins), DIAMOnD algorithm allowed to predict 238 novel putative disease genes. By intersecting these 238 DIAMOnD genes with the 448 AD-specific switch genes, we found a set of 14 genes to be proposed to enlarge the known AD disease module (Fig. 5). The complete list of these 14 genes and their statistics are reported in Table 3. Interestingly, all of them are up-regulated in AD condition (Table 3).

**Table 3:** AD gene signature.

Gene symbol	Gene name	Gene ID	*p* value	FDR	logFC	Direction
AKT1	AKT serine/threonine kinase 1	207	1.05E−05	1.03E−04	0.0093	Up
CBL	Cbl proto-oncogene	867	2.60E−06	3.06E−05	0.0162	Up
CSF3R	Colony stimulating factor 3 receptor	1441	1.23E−08	2.73E−07	0.0228	Up
DOK3	Docking protein 3	79,930	1.50E−05	1.41E−04	0.0113	Up
HCK	HCK proto-oncogene, Src family tyrosine kinase	3055	1.54E−06	1.93E−05	0.0165	Up
INPPL1	Inositol polyphosphate phosphatase like 1	3636	7.24E−07	9.83E−06	0.0182	Up
PRKCD	Protein kinase C delta	5580	6.36E−11	2.45E−09	0.0139	Up
PTPN6	Protein tyrosine phosphatase non-receptor type 6	5777	2.31E−06	2.78E−05	0.0079	Up
STAT3	Signal transducer and activator of transcription 3	6774	1.23 E−04	8.89E−04	0.0153	Up
STAT5A	Signal transducer and activator of transcription 5A	6776	6.02E−05	4.83E−04	0.0125	Up
STAT5B	Signal transducer and activator of transcription 5B	6777	1.26E−07	2.13E−06	0.0174	Up
TNK2	Tyrosine kinase non receptor 2	10,188	2.34E−08	4.84E−07	0.0101	Up
TYK2	Tyrosine kinase 2	7297	4.93E−18	1.01E−15	0.0297	Up
WAS	WASP actin nucleation promoting factor	7454	4.95E−07	7.11E−06	0.0157	Up

AD, Alzheimer’s disease; CTL, control; FC, fold-change AD/CTL; FDR, False Discovery Rate.

Then, following the fundamental principle of Network Medicine stating that the molecular determinants associated with a given disease should be agglomerate in specific regions of the human interactome (disease modules)^4^, we verified that this novel 14-gene signature, together with the seed proteins of AD, actually form a statistically significant disease module (Fig. 5 and Supplementary Figure 2).

## Discussion

In the last few years, the new paradigm of Network Medicine overcame the conventional medicine paradigm ‘one gene, one drug, one disease’ mainly focused on treating the symptoms rather than discovering the causes of diseases^4,5^. According to the Network Medicine paradigm, diseases are rarely caused by a single gene mutation, but more typically by the deregulation of a set of genes interconnected with each other within the human interactome. Moreover, it is becoming increasingly evident that the molecular determinants associated with a given disease (named disease genes) have a high propensity to agglomerate in specific regions of the interactome, suggesting the existence of specific disease network modules for each disease^5–10^. Thus, identifying fully these disease modules and understanding the effects of their perturbations on disease onset and progression could lead to unveil new diagnostic biomarkers as well as therapeutic targets.

Following this innovative vision of medicine, in the present paper, we carried out a computational analysis by integrating consolidated network-medicine tools and concepts in order to unveil novel putative disease genes associated with one of the most common and awful neurodegenerative diseases, i.e., the Alzheimer’s disease. In particular, we exploited the DIAMOnD methodology^12^ combined with the SWIM methodology^15,30^ to enlarge the set of known AD-associated genes (seed) with additional 14 genes having peculiar and crucial characteristics, i.e.:

- they have a significant fraction of their interactions with the seed of AD in the human interactome (Fig. 1);
- they have biological characteristics similar to the seed of AD (Fig. 3);
- they are switch genes that are similarly modulated (all up-regulated in AD) and are important inter-module connectors in the gene co-expression network (Table 3 and Fig. 4);
- they form a statistically significant disease module in the human interactome (Fig. 5 and Supplementary Figure 2).

We finally investigated the current biological knowledge of the 14-gene signature in the AD framework.

The AKT serine/threonine kinase 1 (AKT1) is a key player in the signaling of insulin and other growth factors and its alterations were widely associated with AD pathology and lower cognitive function^37,38^. Indeed, insulin and the PI3K-AKT signaling pathway have a significant role in neuronal health as well as synapse formation and maintenance^39^. Interestingly, enhancement of PI3K-AKT signaling in the central nervous system by intranasal insulin treatments was shown to improve memory in vivo in mice and in human trials^38^. Also, the inositol polyphosphate phosphatase like 1 (INPPL1), which encodes the protein SHIP2, play a key role in the regulation of insulin function and growth factor receptors turnover. The study by Mostafavi et al.^40^ showed that the upregulation of INPPL1 gene significantly correlates with cognitive decline in human AD patients. Other independent studies reported SHIP2 functions as a mediator of amyloid toxicity via tau hyperphosphorylation^41^ and actin-cytoskeleton reorganization^42^.

The Cbl proto-oncogene (CBL) encodes a RING finger E3 ubiquitin ligase, which is an enzyme required for targeting substrates for degradation by the proteasome. Several studies have indicated that CBL functions as a negative regulator of many signal transduction pathways^43,44^, and in particular, it could suppress the Src-family tyrosine kinase Fyn previously identified as potential target for AD^45^. Another member of Src family of tyrosine kinases is the HCK proto-oncogene, Src family tyrosine kinase (HCK). In particular, HCK is a hematopoietic cell kinase whose dysregulation may affect microglia (i.e., resident immune cells in the central nervous system playing critical roles in brain immunity, development, and homeostasis) and accelerate early stage Alzheimer's disease-like neuropathology^46^. Recently, also the colony stimulating factor 3 receptor (CSF3R) and the docking protein 3 (DOK3) was found to be involved in the in microglial cell activation^47,48^.

The protein kinase C delta (PRKCD) encodes one of the protein kinase C family members that are involved in physiological processes related to learning and memory, and thus have been classified as cognitive kinases. Besides their implication in memory and cognition, PKC family members were found to regulate several pathways relevant for AD pathophysiology and thus proposed as potential therapeutic strategy against AD^49^. To our knowledge, there currently exist no studies associating the tyrosine kinase non receptor 2 (TNK2) as well as the WASP actin nucleation promoting factor (WAS) with the etiology and pathogenesis of AD.

Finally, the signal transducer and activator of transcription 3 (STAT3), the signal transducer and activator of transcription 5A (STAT5A) and 5B (STAT5A), and the tyrosine kinase 2 (TYK2) are key components of the JAK-STAT signaling pathway which seems to promote neuroinflammation in neurodegenerative diseases^50^.

## Limitations of the study

One limitation of the DIAMOnD-based analysis is that it assumes that the new candidate disease genes must be topologically, functionally, and biologically close to the starting seed proteins. In fact, the algorithm firstly explores the nearest neighborhood of the seed proteins in the human interactome (topological vicinity) and prioritizes those proteins having a significant fraction of their physical and functional interactions with them (functional vicinity). Then, by using the biological criterion, it selects among them those proteins that have biological characteristics like the ones of the initial seed proteins (biological vicinity). However, the incompleteness of the human interactome provides a partial knowledge of the physical and functional interactions among all the human proteins. Thus, starting from the seed proteins used in^12^, no genes reported by Bellenguez et al.^28^ will be predicted because direct interactions between them are not yet known, and the two lists are now topologically and functionally distant in the human interactome. By increasing the number of DIAMOnD interactions up to 1000, just one protein out of the ones identified by Bellenguez et al.^28^ was found.

## Conclusion

The present study showed how a systematic analysis exploiting tools and concepts of the new paradigm of Network Medicine can improve the prediction of a novel AD gene signature that may contribute to the pathological phenotype. Indeed, our network-based pipeline allowed to complement the current knowledge of AD-associated genes, mainly stemmed from GWAS analysis, with additional putative AD biomarkers, mainly stemmed from measuring transcript abundance and gene expression patterns. It is worth stressing that our study has a purely computational nature and experimental validations would be necessary to investigate the actual role of the identified genes as AD biomarkers. However, we believe that our findings could deepen the understanding of mechanisms underlying AD pathogenesis, maybe suggesting new potential diagnostic biomarkers and/or therapeutic targets for this awful neurodegenerative disease.

## Supplementary Information

Supplementary Information 1.

Supplementary Table 1.

Supplementary Table 2.

Supplementary Table 3.

## Data Availability

Data that support the findings of this study are openly available in GEO public repository and in Supporting Information files of^12^. SWIMmeR code is freely available at https://github.com/sportingCode/SWIMmeR. DIAMOnD code is freely available from the Supporting Information files of^12^. All the other relevant data are within this manuscript and its Supporting Information files.
