# Supplementary material for: Alzheimer’s disease: insights from a network medicine perspective
Source: Sci Rep. 2022 Oct 7;12:16846. doi: 10.1038/s41598-022-20404-3 (PMC9546925; doi:10.1038/s41598-022-20404-3)
Supplement: Supplementary file 1 — Supplementary Information 1. [file 41598_2022_20404_MOESM1_ESM.docx]

**Alzheimer’s disease: insights from a network medicine perspective**

Federica Conte ^1^, Paola Paci ^1,2*^

1. Institute for Systems Analysis and Computer Science “Antonio Ruberti”, National Research Council, Rome, Italy

2. Department of Computer, Control and Management Engineering, Sapienza University of Rome, Italy

*Correspondence: [paci@diag.uniroma1.it](mailto:paci@diag.uniroma1.it)

# Supplementary Information

This document contains the Supplementary Figures and the descriptions of the Supplementary Tables provided as .xlsx files.

# Supplementary Figures

**
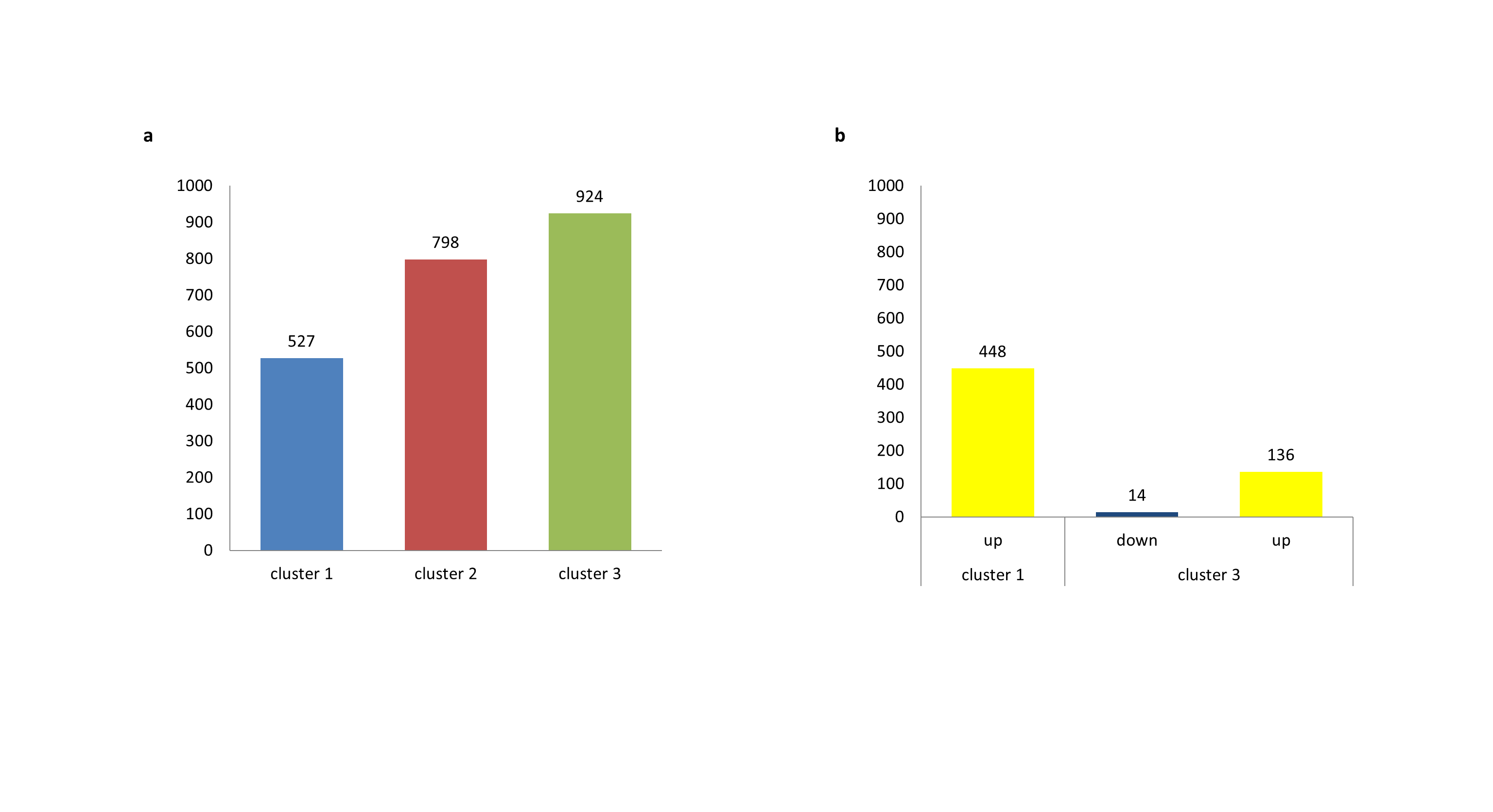
**

**Supplementary Figure 1. SWIMmeR correlation network.** Distribution of the number of the total network nodes (a) and switch genes (b) in the SWIMmeR correlation network.


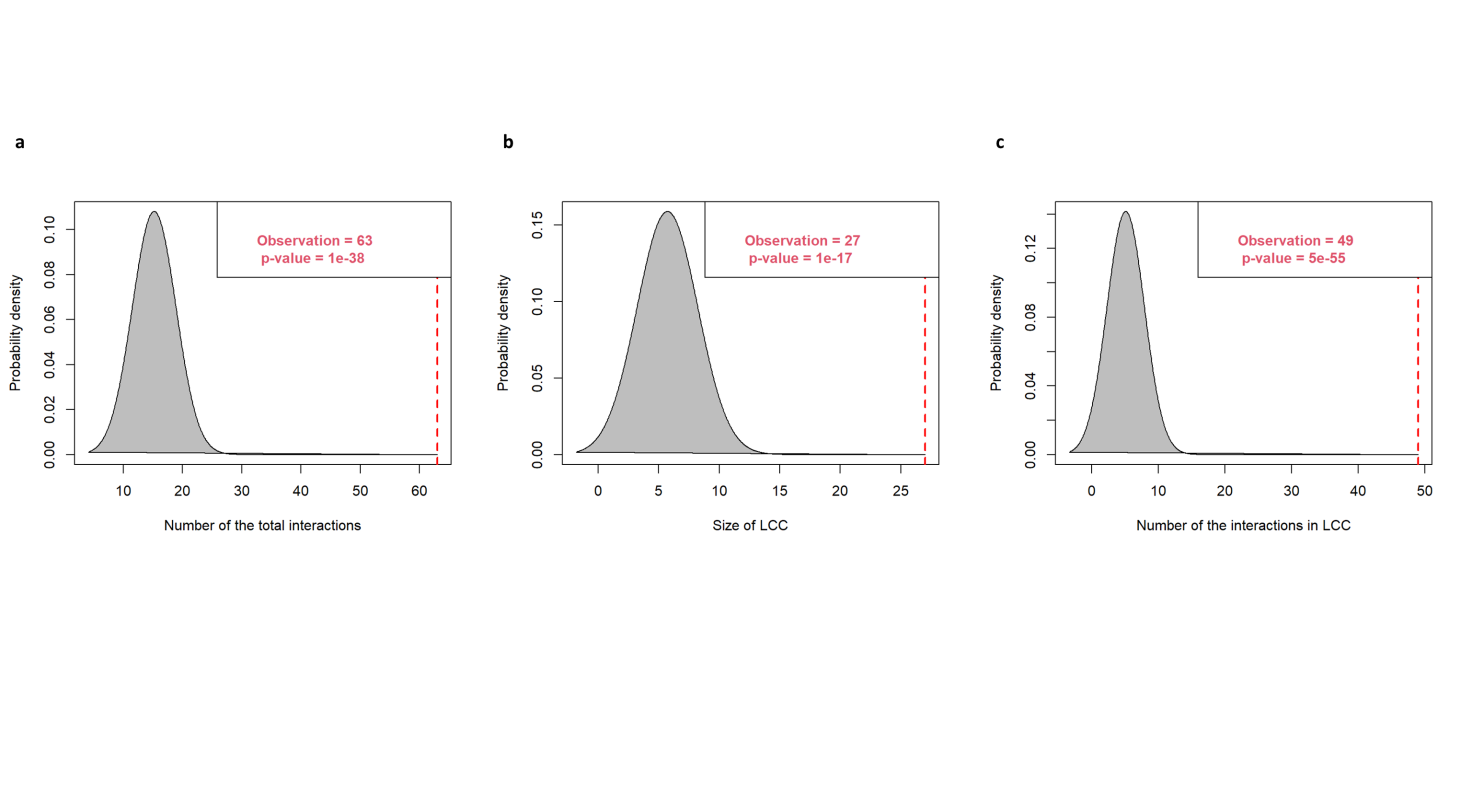


**Supplementary Figure 2. Disease module of AD.** Distribution of the number of total interactions (a), the size of the largest connected component (LCC) (b), and the number of edges in the LCC (c) in the subnetwork induced by a randomly selected gene set of the same size and degree distribution as the original list of genes (99 known AD-associated genes plus 14 switch genes) in the human interactome. Dashed red lines correspond to the observed values of each metric computed for the original list of genes mapped onto the interactome. All p-values were calculated by using a one-tailed z test.

# Supplementary Tables

**Supplementary Table 1.** **DIAMOnD results**. The file is composed of three separated sheets. The first sheet reports the list of known disease genes (seed) associated to AD. The second sheet reports the list of novel putative disease genes predicted by DIAMOnD for AD and their statistics. The third sheet reports the list of the selected DIAMOnD genes.

**Supplementary Table 2**. **SWIMmeR results**. The file is composed of four separated sheets. The first sheet reports the list of the differentially expressed genes computed by SWIMmeR and their statistics for the AD dataset. The second sheet reports the list of the nodes of the SWIMmeR correlation network computed and their statistics for the AD dataset. The third sheet reports the list of the switch genes computed by SWIMmeR and their statistics for the AD dataset. The fourth sheet reports the list of the selected switch genes.

**Supplementary Table 3**. **Functional enrichment analysis of switch genes**. The file is composed of two separated sheets. The first sheet reports the GO Biological Processes found to be enriched in the list of AD switch genes. The second sheet reports the list the DisGeNET diseases found to be enriched in the list of AD switch genes.
